# A Multi-Institutional Survey on Faculty Development Needs, Priorities and Preferences in Medical Education in an Asian Medical School

**DOI:** 10.3885/meo.2009.Res00317

**Published:** 2009-09-23

**Authors:** Zubair Amin, Khoo Hoon Eng, Chong Yap Seng, Tan Chay Hoon, Goh Poh Sun, Dujeepa D Samarasekera, Chan Yiong Huak, Koh Dow Rhoon

**Affiliations:** Yong Loo Lin School of Medicine, National University of Singapore, 10 Medical Drive, Singapore 117590, Singapore

**Keywords:** faculty development needs assessment, Singapore

## Abstract

**Background:**

Faculty development in medical education is crucial for maintaining academic vitality. The authors conducted a needs assessment survey in Singapore to determine the educational needs and priorities of clinical faculty.

**Methods:**

This study implemented a questionnaire-based, anonymous, multi-institutional survey with stratified random sampling. Each question was anchored with two statements on a 9-point scale. Respondents were asked to determine their current knowledge and the knowledge they would need in future.

**Results:**

The response rate was 81.9%. Overall, the participants’ current knowledge was rated either “modest” (scale 4-6) or “substantial” (scale 7-9), irrespective of teaching experience. Participants reported higher knowledge in areas related to teaching and modest knowledge in educational concepts and assessment. They reported a need for higher knowledge in most areas to function well as a teacher.

**Conclusion:**

The need for faculty development is universal and independent of teaching experience in this group. Teaching faculty from the institutes studied understood the need for improved knowledge in pedagogical knowledge.

Faculty development in education is a constellation of planned activities, designed to improve and enhance faculty members’ knowledge and skills as teachers. Generally, these include the domains of teaching, assessment, curriculum support, organizational leadership, and mentoring. Faculty development is viewed as outward signs of inner faith that the institutions have in their workforce.^1^ A comprehensive faculty development program ensures that the formal curriculum prescribed is actually delivered, as we know that the hidden curriculum, the true interface between teaching and learning and teachers and learners, is powerful and has more lasting consequences than the formal curriculum.

Medical schools around the world have embarked on some form of curriculum renewal and introduced educational innovations.^2^ Faculty development helps ensure that the educational reforms and initiatives are worthy and implemented properly. Professional organizations and experts advocate greater awareness and acquisition of knowledge in teaching and learning through comprehensive faculty development.^3^,^4^

Faculty development is a reflective process that includes deliberate introspection, determination of one's own needs and demands of the work, identification of the gaps, and taking actions. The realization of the *gap*, the difference between required knowledge and current knowledge, is frequently the primary motivating factor towards pursuing further training in pedagogy. From an institutional perspective, realizing the gap is essential for better planning and more efficient resource allocation.

In order to determine educational needs of the clinical faculty and to identify the priority-areas of faculty members’ educational knowledge in Yong Loo Lin School of Medicine (YLLSoM), National University of Singapore (NUS), we conducted a multi-institutional survey. We hoped that data would help us not only to plan a comprehensive faculty development program based on our needs and educational mission but would also support the educational reform within the school.

## Methods

**Context** – Our school of medicine's undergraduate curriculum is broadly divided into 2 phases: pre-clinical (Years 1 and 2) and clinical (Years 3 to 5). The students in clinical years rotate through several large affiliated teaching hospitals and institutes in Singapore. Teaching in these hospitals is mostly carried out by clinicians under a “Clinical Faculty Scheme”. This number excludes full-time academic clinicians employed by the university. YLLSoM conducts faculty development programs on basic pedagogical principles, problem-based learning, clinical teaching, student assessment, mentoring, and educational leadership. Some affiliated hospitals conduct their own faculty development programs.

**The questionnaire** – We developed the questionnaire through a multi-phase process. First, we identified areas relevant to our clinical faculty (e.g., teaching and learning concepts, educational methods, assessment) through focus group discussions. Second, we reviewed literature on faculty development and selected items that were of importance.^4^–^8^ Third, we used our own contextual knowledge about the educational ecosystem in Singapore. The preliminary questionnaire was further reviewed and pilot tested (see Table 1).

**Table 1.** Questionnaire items and sub-categories

Items Used in Questionnaire
Educational Concepts
Emerging Trends and Issues
Teaching and Learning Concepts
Course and Module Design
Educational Objectives
Educational Strategies
Teaching and Learning Strategies
Lecture and Large Group Teaching
Tutorial and Small Group Teaching
Teaching Communication and Counseling Skills
Bedside and Clinical Teaching
Facilitating Problem-Based Learning
Feedback
Use of IT and Computer in Education
Assessment
Assessment Concepts
Selecting an Assessment Instrument
Assessment of Knowledge Using Essay and Modified Essay Question
Assessment Using MCQ^*^
Assessment Using OSCE^†^
Assessment of Professional Behavior
Teaching House Officers and Medical Officers
Educational Resources

^*^MCQ: Multiple choice questions.

^†^OSCE: Objective structured clinical examination.

We used a 9-point anchored scale. For each item there were two anchor statements. The first statement described someone who did not have even basic knowledge of the topic. The second statement referred to someone who has significant knowledge in the particular area and was able to apply the knowledge.

We defined scale points 1 to 3 as ‘Limited Knowledge’, 4–6 as ‘Modest Knowledge’, and 7–9 as ‘Substantial Knowledge.’ For each item we asked the participants to identify what their current knowledge was and what they believed their future knowledge should be. Participants opted for option ‘0’ and left out that particular item if the topic was irrelevant to their present and future work.

The demographic section asked information about the nature of activities in which respondents were involved (teaching in large class, tutorials, clinical teaching, laboratory teaching, and others) and the number of years involved in student assessment (≤3 yrs, 4–9 years, 10–19 years, and ≥20 years). Several other investigators used similar instruments in faculty development needs assessment surveys.^9^,^10^

**Properties of the questionnaire** – Our approach to establishing validity was a judgmental process as opposed to an empirical data-driven approach, which is more applicable to determining predictive and concurrent validity. The content validity of the instrument was determined by the representation and relevance of topics to the target group.^4^,^7^,^8^ Reliability, as determined by internal consistency, was found to be high (Cronbach alpha > 0.90) in a prior study with a similar instrument.^5^

**Sample size determination** – The total number of clinicians under the clinical faculty scheme was 868. From a pilot survey, we ascertained that at least 80% of the clinicians wanted to have better knowledge by 3 points. We assumed, conservatively, a similar profile of response. We calculated that we would need 218 clinicians to be sampled to reach a confidence interval of 75–85% and to extrapolate the study findings to the larger group. Assuming the response rate would be 80%, we would therefore need to send the questionnaire to 272 respondents, i.e., a little over 30% of the target population.

A computer-generated stratified random sample was created from the master list of clinical teachers. There were four stratified groups: a) senior doctors (clinical professor, clinical associate professor, and senior lecturers), b) clinical lecturers, c) clinical teachers, and d) clinical tutors (see Table 2). The distinction between these groups was based on teaching experience and, to a limited extent, quality of teaching and educational scholarship.

**Table 2.** Breakdown of the stratified random sample

	Total number in clinical faculty	Sampled number
Senior doctors	101	33 (33%)
Clinical lecturers	138	53 (38%)
Clinical teachers	355	114 (32%)
Clinical tutors	274	72 (26%)
Total	868	272 (31.3%)

**Data collection, recording, and quality control** – The survey was administered by a research coordinator in paper-and-pencil format. Invitation letters with instructions and ethical considerations were sent to potential respondents to attend a session. Most of the surveys were completed during these sessions. The research coordinator manually collected the survey forms, kept a log, and entered the data. An independent quality check ascertained >99% accuracy.

**Statistical analysis** – We used descriptive statistics to calculate rate, proportion, and ratio. Where the data were not normally distributed, we used the non-parametric Wilcoxon-Signed Ranked test to determine the difference between current and desired knowledge. We used the Chi-square test to determine difference between variables, such as whether the response to a particular item differed between hospitals or between grades.

We used the McNemar test to determine whether those respondents who reported a limited knowledge or modest knowledge wanted their knowledge to be in a higher level (modest or substantial level). The McNemar test is a non-parametric test used to determine differences between two dependent ‘responses’ for a given stimulus. In this study, the stimulus was the question and the two responses were the respondents’ perceived current and desired knowledge. A statistically significant p-value would indicate that there was a change in either a positive (a greater score in ‘desired’ than ‘current’) or negative direction (a lesser score in ‘desired’ than ‘current’). In our analysis, the change in direction was positive, indicating that the respondents wanted improvement.

**Ethical review** – The survey was anonymous. Confidentiality of information collected was maintained throughout all phases of study. Only group data were presented. This study was approved by the Institutional Review Board (IRB, 04.14 E) and supported by the Medical Education Unit of YLLSoM, NUS.

## Results

There were 223 valid responses (81.9% response rate).

**Demographics** – 209 respondents (93.7%) indicated the length that they have been involved in teaching medical students. The distribution was as follows: ≤3 yrs 4%, 4-9 years 45.3%, 10–19 years 35.4%, and ≥20 years 9%. Thirty-five percent of the respondents were involved in teaching large classes, 81.2% in tutorials, 87.9% in clinical teaching, and 2.7% in laboratory-based teaching. The total percentage exceeds 100% as individual respondents were involved in more than one teaching modalities.

**Current and desired knowledge: global analysis** – The mean and median points of participants are shown in Table 3. Overall, the participants reported their current knowledge as either “modest” or at the lower end of the “substantial” level. Participants reported higher current knowledge in areas related to teaching such as lecture and large group teaching, tutorials and small group teaching, teaching communication and counseling, bedside and clinical teaching, and teaching house officers and medical officers. The participants’ need for further knowledge was also higher (median 8.0) in these areas. Conversely, participants reported modest knowledge in areas related to educational concepts and assessment (Table 3). Objective structured clinical examination (OSCE) and assessment of professional behavior were two items where participants wanted much higher knowledge (median 8.0; p < 0.001). For all items, the difference between desired and current knowledge was statistically significant (p < 0.001).

**Table 3.** Current and desired knowledge: global analysis

	Current		Desired	
		
Assessment Topics	Mean (± 1 SD)	Median	Mean (± 1 SD)	Median
Emerging issues and trends (n_1_ = 221; n_2_ = 219)	4.9 (± 1.5)	5.0	7.1 (± 1.3)	7.0
Teaching and learning concepts (n_1_ = 222 n_2_ = 219)	5.0 (± 1.7)	5.0	7.2 (± 1.3)	7.0
Course and module design (n_1_ = 222; n_2_ = 219)	4.4 (± 1.8)	4.0	6.8 (± 1.5)	7.0
Educational objectives (n_1_ = 223; n_2_ = 220)	5.6 (± 1.7)	6.0	7.2 (± 1.3)	7.0
Teaching and learning strategies (n_1_ = 222; n_2_ = 219)	5.7 (± 1.8)	6.0	7.4 (± 1.1)	8.0
Lecture and large group teaching (n_1_ = 223; n_2_ = 220)	6.3 (± 1.6)	7.0	7.6 (± 1.1)	8.0
Tutorials and small group teaching (n_1_ = 223; n_2_ = 220)	6.5 (± 1.5)	7.0	7.7 (± 1.0)	8.0
Teaching communication & counseling (n_1_ = 223; n_2_ = 221)	6.1 (± 1.7)	6.0	7.7 (± 1.1)	8.0
Bedside/clinical teaching (n_1_ = 222; n_2_ = 221)	6.9 (± 1.3)	7.0	7.9 (± 1.1)	8.0
Facilitating problem-based learning (n_1_ = 221; n_2_ = 219)	5.5 (± 1.9)	6.0	7.4 (± 1.3)	8.0
Feedback (n_1_ = 222; n_2_ = 220)	5.8 (± 1.6)	6.0	7.4 (± 1.1)	7.0
Use of IT and computer in education (n_1_ = 222; n_2_ = 221)	5.1 (± 1.9)	5.0	7.3 (± 1.3)	7.0
Assessment concepts (n_1_ = 221; n_2_ = 218)	5.2 (± 1.7)	6.0	7.3 (± 1.2)	7.5
Selecting assessment instrument (n_1_ = 222; n_2_ = 219)	5.0 (± 1.7)	5.0	7.2 (± 1.4)	7.0
Assessment of knowledge using essay and modified essay question (n_1_ = 222; n_2_ = 220)	5.1 (± 1.8)	5.0	7.1 (± 1.4)	7.0
Assessment using MCQ (n_1_ = 222; n_2_ = 220)	5.3 (± 1.8)	6.0	7.2 (± 1.4)	7.0
Assessment using OSCE (n_1_ = 221; n_2_ = 219)	5.3 (± 2.0)	5.0	7.3 (± 1.5)	8.0
Assessment of professional behavior (n_1_ = 222; n_2_ = 220)	5.9 (± 1.9)	6.0	7.6 (± 1.3)	8.0
Teaching house officers & medical officers (n_1_ = 222; n_2_ = 220)	6.3 (± 1.7)	7.0	7.8 (± 1.1)	8.0
Educational resources (n_1_ = 222; n_2_ = 220)	5.8 (± 1.7)	6.0	7.7 (± 1.0)	8.0

n_1_=valid response for current knowledge; n_2_=valid response for desired knowledge.

Scores of current and desired knowledge are statistically significant for all topics (p < 0.001); Wilcoxon Signed Rank test.

We analyzed the difference between desired and current knowledge within individual hospitals and by length of experience. For all items, the difference between desired and current knowledge was statistically significant for both groups (p < 0.05).

We also determined whether the need to improve knowledge in various topics was pedagogically meaningful by grouping the responses into limited, modest, and substantial levels. In all topics except facilitation of problem-based learning (PBL), teaching house officers and medical officers, and educational resources, participants who reported their current knowledge at either the limited or modest levels expressed a need to improve their knowledge to a higher level. For all topics except the three mentioned above, this was statistically significant (p < 0.001).

**Sub-group analysis** – Comparison between desired and current knowledge among faculty with different lengths of teaching showed statistically significant differences (p < 0.05) in the following areas: facilitation of PBL, use of IT and computers in education, assessment concepts, selection of assessment instruments, assessment of knowledge using essay and modified essay questions, assessment of professional behavior, and teaching house officers and medical officers. In these areas, faculty with longer teaching experience reported better knowledge and reported less need for improvement as compared to faculty with shorter teaching experience. Comparison of the differences between current and desired knowledge among the hospitals showed no statistical difference (p > 0.05).

## Conclusions

In our study cohort, the need for faculty development was universal and irrespective of length of teaching experience. It also suggests that the teaching faculty require better knowledge in pedagogy to function properly as teachers.

Participants reported better knowledge in teaching-related items such as lecture and large group teaching, tutorials, teaching communication and counseling, bedside and clinical teaching compared to items that were more theory-based, such as educational concepts or topics related to assessment. These findings are not surprising, as there are many faculty development programs available for participants in teaching-related areas. Moreover, these are activities the participants perform on a daily basis. Intriguingly, they wanted higher knowledge in teaching-related items, indicating their willingness to improve further in relevant areas. In assessment-related items, participants wanted much higher knowledge about OSCEs and assessment of professional behavior. This can be explained by our relatively recent introduction of the OSCE and emphasis on professional behavior.

Our study highlights the importance of context and relevance in faculty development^11^–^13^ Context and relevance in this study can be viewed as the environment where teaching and learning take place and where the actual curriculum is delivered. Participants’ need for knowledge was more noticeable in areas relevant to their current and future works, an important point to consider in an organization with multiple missions and finite resources.

We believe the strengths of this current study are the following: (a) broad sampling and representation (b) high response rate (c) sound psychometric properties of the questionnaire and (d) rigorous monitoring of data quality. We also believe that the findings of the study would be applicable to many other medical schools.

The limitations are related to inherent properties of a questionnaire based self-reported surveys, namely response and social desirability biases. How the perceived knowledge relates to participants’ actual tacit knowledge and practice is not answered from this survey. We encourage institutes to conduct their own survey, based on their own contexts and needs, and not to accept the study findings uncritically.

Our survey findings helped us design our faculty development programs both in terms of content (what we offer) and format (how we offer). We have added new faculty development programs to our existing list to include assessment and educational leadership. Our programs now target physicians with differing experience.

Further extension of this study could include exploring the relationship between the reported and actual knowledge in pedagogy and determining the relationship between respondents’ knowledge and their actual teaching performance.

## Prior Presentation

Data from this survey were presented as an Oral Presentation at the Association of Medical Education in Europe Annual Conference 2006.
